# Mental Health Consequences for Healthcare Workers During the COVID-19 Pandemic: A Scoping Review to Draw Lessons for LMICs

**DOI:** 10.3389/fpsyt.2021.602614

**Published:** 2021-01-27

**Authors:** Modhurima Moitra, Muhammad Rahman, Pamela Y. Collins, Fatima Gohar, Marcia Weaver, John Kinuthia, Wulf Rössler, Stefan Petersen, Jurgen Unutzer, Shekhar Saxena, Keng Yen Huang, Joanna Lai, Manasi Kumar

**Affiliations:** ^1^Department of Global Health, University of Washington, Seattle, WA, United States; ^2^Department of Health Metrics Sciences, University of Washington, Seattle, WA, United States; ^3^Department of Psychiatry and Behavioral Sciences, University of Washington, Seattle, WA, United States; ^4^United Nations International Children's Emergency Fund (UNICEF), New York City, NY, United States; ^5^Kenyatta National Hospital, Nairobi, Kenya; ^6^Department of Psychiatry, Psychotherapy and Psychosomatics, University Hospital of Psychiatry, University of Zurich, Zurich, Switzerland; ^7^Department of Women's and Children's Health, Uppsala University, Uppsala, Sweden; ^8^Department of Global Health and Population, Harvard T H Chan School of Public Health, Harvard University, MA, United States; ^9^Department of Population Health & Child and Adolescent Psychiatry, New York University, New York City, NY, United States; ^10^Department of Psychiatry, University of Nairobi, Nairobi, Kenya; ^11^Department of Clinical, Educational and Health Psychology, University College London, London, United Kingdom

**Keywords:** COVID-19, healthcare worker, mental health conditions, global health, depressive symptoms, anxiety symptoms, distress

## Abstract

**Background:** The COVID-19 pandemic has had a significant impact on the mental health of healthcare workers (HCWs) particularly in low and middle-income countries (LMICs). This scoping review provides a summary of current evidence on the mental health consequences of COVID on HCWs.

**Methods:** A scoping review was conducted searching PubMed and Embase for articles relevant to mental health conditions among HCWs during COVID-19. Relevant articles were screened and extracted to summarize key outcomes and findings.

**Results:** A total of fifty-one studies were included in this review. Depressive symptoms, anxiety symptoms, psychological trauma, insomnia and sleep quality, workplace burnout and fatigue, and distress were the main outcomes reviewed. Most studies found a high number of symptoms endorsed for depression, anxiety, and other conditions. We found differences in symptoms by sex, age, and HCW role, with female, younger-aged, frontline workers, and non-physician workers being affected more than other subgroups.

**Conclusion:** This review highlights the existing burden of mental health conditions reported by HCWs during COVID-19. It also demonstrates emerging disparities among affected HCW subgroups. This scoping review emphasizes the importance of generating high quality evidence and developing informed interventions for HCW mental health with a focus on LMICs.

## Introduction

From January to August 2020, over 800,000 people have died due to the COVID-19 pandemic, and health departments confirmed more than 24 million cases worldwide (1). The high transmission rate of SARS-CoV-2 and the severity of illness associated with COVID-19 have severely strained healthcare systems globally. The media and researchers continue to monitor and report the availability of hospital space, equipment, and treatment supplies (2). Shortages of personal protective equipment (PPE) and ventilators have been widely discussed in the literature (3, 4). The World Health Organization (WHO) has also issued guidance around managing such shortages by recommending reasonable and appropriate usage of PPE (5).

An equally important issue is the severe threat to the well-being of healthcare workers (HCWs) during this pandemic. HCWs currently comprise the most critical sector of the workforce at the frontlines of testing and treatment for COVID-19 as well as covering other essential health services. They are at an unusually high risk of exposure to infection, and they are also at high risk of developing mental and behavioral disorders due to the high psychological toll of their intensive work in managing this highly infectious virus and grieving deaths of their colleagues (6, 7). Several studies and reviews have examined the psychological effects of the COVID-19 pandemic on HCWs (8, 9). HCWs experience symptoms of depression and anxiety, sleep problems, burnout, and general psychological distress (10–12). Understandably, at this stage in the pandemic existing studies show considerable variation in sampling methods and measurement of mental health outcomes. Nevertheless, emerging differences in mental disorder prevalence by sex and worker type may be of public health significance and warrant closer review (12).

Although HCWS across the world are currently overwhelmed by the demands of care for COVID-19 patients, the impact of the pandemic on the mental health of HCWs holds significance for low and middle-income countries (LMICS) in particular. Health systems in LMICs typically do not effectively incorporate continued care modalities for chronic conditions in comparison to high-income countries (HICs). Mental healthcare services for HCWs in particular are also far less accessible in LMICs than in HICs. The onset of COVID-19 has now resulted in a tremendous increase in demand for care in health systems that are already taxed with a higher burden of acute care compared to HICs. Adequate equipment and infrastructure to treat those with complex and intensive care needs are limited in supply. Therefore, LMICs have to navigate a complex trade-off—preventing transmission, managing existing cases, as well as keeping their health systems and economies on track. Given these challenges LMICs face, supporting the well-being of HCWs is essential to maximizing benefits to those who need their services in both the short and long term.

Given the rapidly evolving nature of the evidence, we provide an update of the current evidence base on HCW mental health in LMICs. We review evidence from all locations but use our findings to guide recommendations for LMICs. This scoping review aims to (a) summarize emerging themes and results from key quantitative and qualitative evidence published between December 2019 and June 2020 on the psychological consequences of COVID-19 on HCWs, and (b) use the updated evidence base to provide a set of broad recommendations for mental health support for HCWs in LMICs. Findings highlighted here may guide the implementation of workplace interventions in low-resourced healthcare settings and help policymakers and health ministries develop mitigation policies and programs supporting the mental health of HCWs.

## Methods

### HCW Case Definition

For the purpose of this scoping review, we adhered to the Centers for Disease Control and Prevention (CDC) definition of HCWs which includes physicians, nurses, emergency medical personnel, dental professionals and students, medical and nursing students, laboratory technicians, pharmacists, hospital volunteers, and administrative staff (13).

### Search Strategy

**Figure 1:**
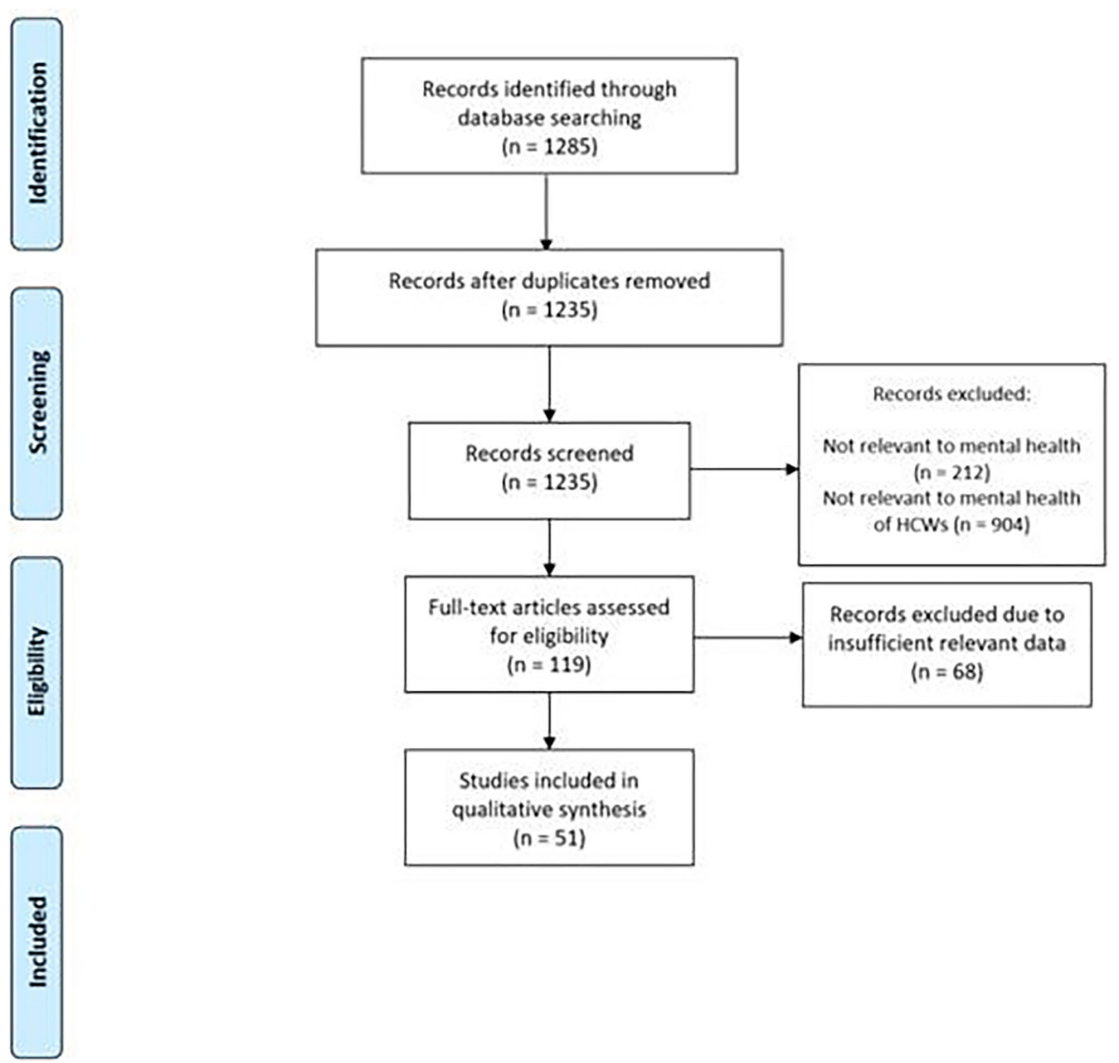
PRISMA Flowchart of Study Selection.

A scoping review was conducted using PubMed and Embase (Dec 2019–June 2020) to broadly search for studies relevant to mental health in the context of COVID-19. Details of the search strings are in the Appendix. Papers on mental health conditions among HCWs were then screened and selected for this review. According to out inclusion criteria, papers were included if they reported any quantitative or qualitative data on mental disorders or symptoms and other related conditions among healthcare workers such as psychological trauma, distress, and workplace burnout. According to our exclusion criteria, papers were excluded if they did not provide pertinent information on mental health outcomes among HCWs or were not relevant to mental health during COVID-19. Selected studies were screened and information on study characteristics and outcomes reported were extracted. Findings from our review were then summarized by study methodology, outcomes, and HCW group in the results section below. MM, MR, and MK screened studies. MM and MR extracted data from selected studies. Any questions around study eligibility were jointly discussed and resolved by MM, MR, and MK. MM summarized findings from the final list of studies. The PRISMA study selection flowchart is provided in Figure 1.

## Results

A total of 51 studies were selected for this review. Table 1 shows the countries represented in these studies. A majority, 61% of selected studies, originated from China. Based on World Bank Income level classifications, 72.5% of the studies were from Low and Middle Income Countries (LMICs). The sample size of included studies ranged from 52 to 14825 with a median sample size of 548. Of the 39 studies that reported data on the distribution of HCWs by sex, the median proportion of female HCWs was 64%. However, more than half the sample comprised female HCWs in 79% of these studies. All HCWs (physicians, nurses, and other workers) were represented in 78% of studies. Approximately 6% of studies reported exclusively on non-physician samples (example nurses, therapists) and 16% reported exclusively on physicians. Frontline or first-line medical workers (FMW), who typically are first responders to COVID-19 crises, are mentioned in the sample descriptions of nine studies.

**Table 1:** List of Selected Studies and Study Characteristics.

**References**	**Country**	**World bank income level**	**Healthcare worker sample description**	**Mental health conditions/stressors included**
Alhaj et al. (14)	Multi-country: Canada, the United States, Kuwait, Saudi Arabia, Serbia, and Italy	High income and upper middle income	Neurosurgery residents	Mental health, workplace stress (lack of social life)
Amerio et al. (15)	Italy	High income	General practitioners	Depressive symptoms, anxiety symptoms, insomnia, distress
Cai et al. (16)	China	Upper middle income	Healthcare workers who provided treatment during outbreak	Mental health
Chen et al. (17)	China	Upper middle income	Doctors and nurses	Depressive symptoms, anxiety symptoms, distress, insomnia
Chen et al. (18)	China	Upper middle income	Medical workers	Mental health
Chen et al. (19)	Taiwan	High income	Healthcare workers in a tertiary referral center	Mental health
Chowdhury et al. (20)	United Kingdom	High income	Physicians, nurses, and other professionals	Depressive symptoms, anxiety symptoms, distress, workplace burnout
Chung and Yeung (21)	China	Upper middle income	All healthcare workers	Depressive symptoms
Civantos et al. (22)	United States	High income	Otaloaryngology physicians and residents	Depressive symptoms, anxiety symptoms, distress, workplace burnout
Dimitriu et al. (23)	Romania	High income	First-Line medical residents in emergency unit, ICU, radiology, general surgery, gynecology, and orthopedics	Workplace burnout
Du et al. (24)	China	Upper middle income	Frontline workers	Depressive symptoms, anxiety symptoms, distress, sleep quality
Fawaz and Samaha (25)	Lebanon	Upper middle income	Nurses and physicians caring for COVID patients	Anxiety symptoms (worry related to infection)
Feng et al. (26)	Taiwan	High income	Nurses	Anxiety symptoms (worry related to infection, caring for others, social isolation)
Fernandez et al. (27)	Spain	High income	Physicians, nurses, and other professionals	Anxiety symptoms, depressive symptoms, distress
Guo et al. (28)	China	Upper middle income	Physicians, nurses, medical students, medical assistants, administrative positions, frontline workers	Anxiety symptoms, depressive symptoms
Huang and Zhao (29)	China	Upper middle income	Doctors, nurses, and health administrators	Depressive symptoms, anxiety symptoms, and sleep quality
Huang et al. (30)	China	Upper middle income	First-Line medical staff	Anxiety symptoms, psychological trauma symptoms
Khanna et al. (31)	India	Lower middle income	Ophthamologists and ophthamology trainees	Depressive symptoms
Khusid et al. (32)	United States	High income	Urology residents	Anxiety symptoms, depressive symptoms
Lai et al. (33)	China	Upper middle income	Healthcare workers in fever clinics or COVID wards	Anxiety symptoms, depressive symptoms, distress, insomnia
Li et al. (34)	China	Upper middle income	First-Year training physicians	Anxiety symptoms, depressive symptoms, mood valence, workplace violence
Liang et al. (35)	China	Upper middle income	Doctors and nurses	Depressive symptoms, anxiety symptoms
Liu et al. (36)	China	Upper middle income	Medical staff	Anxiety symptoms
Lu et al. (37)	China	Upper middle income	Doctors, nurses, and administrative staff	Anxiety symptoms, depressive symptoms
Morgantini et al. (38)	60 countries including Brazil, Sweden, Italy, and USA	Various including high and upper middle income	Healthcare professionals	Workplace burnout
Ni et al. (39)	China	Upper middle income	Community-based adults and healthcare professionals	Anxiety symptoms
Poddar et al. (40)	India	Lower middle income	Dermatologists and non-dermatologists (internists, pediatricians, otorhinolaryngologists, respiratory medicine specialists, psychiatrists, and general physicians)	Perceived stress
Qi et al. (41)	China	Upper middle income	Frontline medical workers	Depression and anxiety symptoms, insomnia, sleep quality
Liu et al. (42)	China	Upper middle income	Physicians and nurses	Anxiety symptoms, burnout
Ramaci et al. (43)	Italy	High income	Doctors and nurses	Workplace burnout and fatigue, self-worth, fear
Rossi et al. (44)	Italy	High income	Healthcare workers	Anxiety symptoms, depressive symptoms, distress, insomnia, psychological trauma
Salman et al. (45)	Pakistan	Lower middle income	Medical doctors, nurses, pharmacists	Anxiety symptoms, depressive symptoms, coping strategies
Shariati et al. (46)	Iran	Upper middle income	Psychiatric trainees	Distress
Sheng et al. (47)	China	Upper middle income	Nursing interns	Anxiety symptoms, depressive symptoms, sleep quality
Song et al. (48)	China	Upper middle income	Medical staff in emergency departments	Depressive symptoms, psychological trauma symptoms
Sun et al. (49)	China	Upper middle income	Nurses	Distress
Temsah et al. (50)	Saudi Arabia	High income	Physicians, interns, nurses, midwives, auxiliary services in acute care, general care, auxiliary services, outpatient clinics, and academic units	Anxiety symptoms
Tian et al. (51)	China	Upper middle income	Frontline health professionals	Anxiety symptoms, depressive symptoms, distress, insomnia severity
Wang et al. (52)	China	Upper middle income	Pediatric healthcare workers	Anxiety symptoms, depressive symptoms, sleep quality
Zhang et al. (53)	China	Upper middle income	Medical and non-medical healthcare workers	Anxiety symptoms, depressive symptoms, insomnia
Wu et al. (54)	China	Upper middle income	Medical staff and college students	Distress
Wu and Wei (55)	China	Upper middle income	Frontline medical staff	Anxiety symptoms, depressive symptoms, insomnia, mental health status
Xing et al. (56)	China	Upper middle income	Medical personnel	Medical personnel
Xu et al. (57)	China	Upper middle income	Surgical medical staff	Anxiety symptoms, depressive symptoms
Yang et al. (58)	South Korea	High income	Physical therapists	Anxiety symptoms, depressive symptoms
Yin et al. (59)	China	Upper middle income	Frontline healthcare workers	Sleep quality, psychological trauma symptoms
Zhang et al. (60)	Iran	Upper middle income	Depressive symptoms, distress, anxiety symptoms, job satisfaction	Doctors, nurses, radiologists, technicians
Zhang et al. (61)	China	Upper middle income	Hospital staff	Anxiety symptoms, depressive symptoms, insomnia
Liu et al. (62)	China	Upper middle income	Doctors and nurses	Anxiety symptoms, depressive symptoms, distress
Zhou et al. (63)	China	Upper middle income	Frontline medical workers	Distress, mental health status, sleep quality
Zhu et al. (64)	China	Upper middle income	Doctors, nurses, and medical technicians	Anxiety symptoms. Depressive symptoms, distress

The studies predominantly made use of a cross-sectional study design (88% of studies). Nearly all studies used a convenience sampling strategy and online data collection methods. Symptoms of anxiety (65% of studies), depression (57% of studies), and distress (37% of studies) were the most commonly studied conditions. The Patient Health Questionnaire (PHQ) (33% of studies) and the Generalized Anxiety Disorder Scale (GAD) (29% of studies) were the most commonly used instruments to measure symptoms of depression and anxiety, respectively.

### Anxiety Symptoms

HCWs frequently reported symptoms of anxiety (14, 39, 47, 50, 57, 60). Anxiety was frequently associated with lowered sleep quality and insomnia and was common among those living with infants or elderly family members (32, 47, 58, 61). Severe anxiety was associated with being in direct treatment contact with COVID-19 and the lack of adequate personal protective equipment (PPE) (32, 36). Differences by age were also reported, with younger HCWs experiencing higher levels of anxiety compared to older age groups (30, 44, 45). Differences between male and female HCWs were also found, with women experiencing more symptoms of anxiety compared to men (20, 22, 24, 30, 32, 44, 45, 64). Studies that examined mental health outcomes before and during the COVID-19 outbreak found a significant increase in reported anxiety symptoms in the outbreak period compared to the non-outbreak period (19, 34, 57).

Differences by HCW role: Differences in anxiety symptoms were also reported by HCW role and proximity to patients with COVID-19. In a study examining mental health conditions among physicians, nurses, and other professionals, nurses, and trainees reported higher anxiety than physicians (27). Similar findings are reported elsewhere with HCWs in non-physician roles such as nurses experiencing greater anxiety than those in physician roles (20, 29, 62, 64). Front-line medical workers who are typically required to respond to COVID-19 emergencies experience higher levels of anxiety compared to non-frontline HCWs (28, 51, 55). HCWs in patient care roles experience higher anxiety than those in non-patient care roles or administrative roles (37, 53). Similarly, those working in ICU wards reported greater anxiety compared to those who worked in isolation wards or other departments (45).

### Depressive Symptoms

Symptoms of depression are highly prevalent among HCWs. Similar to anxiety symptoms, depressive symptoms were also associated with poor sleep quality and insomnia (47, 52, 61). Moderate to severe depressive symptoms were also linked to higher access to COVID information, lack of adequate PPE, and treating more COVID-19 patients (15). Studies that prospectively evaluated mental health outcomes found a significant increase in symptoms of depression in the outbreak period compared to the non-outbreak period (19, 34, 57).

Several studies showed that younger age was associated with a higher number of reported depressive symptoms (31, 35, 44, 45, 48). Female HCWs reported experiencing a higher severity of depressive symptoms than males in several studies that examined differences by sex (24, 31–33, 44, 45, 64) with the exception of one study that reported higher depressive symptom severity in male HCWs compared to female counterparts (20). A study of otolaryngology physicians found no sex differences in depressive symptoms among HCWs (22).

Reported depressive symptoms also differed by healthcare roles and level of professional experience. A prospective cohort of first-year trainee physicians in China reported significant increases in depressive symptom severity during the COVID-19 outbreak (34). Nurses and other HCWs in non-physician roles experienced greater depressive symptom severity compared to HCWs in physician roles (20, 27, 28, 33, 62, 64). Those in government health services reported more depressive symptoms compared to other sectors such as private practice or NGOs (31). Proximity to COVID-19 crises also affect depression severity (45). A study of frontline medical workers (FMWs) in China found that over 45% of FMWs experienced symptoms of depression (51). Several studies showed that FMWs reported greater depressive symptom severity when compared to non-FMWs (28, 33, 55). Similarly, HCWs in medical roles experience greater depression symptom severity than those in non-medical roles in healthcare (53).

### Insomnia and Sleep Quality

Insomnia and poor sleep quality were commonly reported by HCWs alongside depression, anxiety and stress (15, 17, 33, 47, 59). Similar to depression and anxiety, there are differences that emerged by sex and occupational role. A study conducted among hospital staff in China between January and February 2020 showed that females reported higher rates of insomnia compared to males (61). The same study also found that nurses experienced greater insomnia severity (ascertained using the Insomnia Severity Index–which measures the severity and frequency of the sleep disorder insomnia) compared to doctors and other medical staff (61). Similar findings were also reported in a study of mental health outcomes among HCWs in Italy: nurses and healthcare assistants reported greater insomnia severity than physicians, general practitioners and other medical professions (44). Proximity to COVID-19 cases was also associated with poor sleep quality (ascertained using the Pittsburgh Sleep Quality Index–which measures recent deviances in usual sleeping habits) and insomnia. Frontline workers and medical HCWs experienced worse sleep quality and higher insomnia severity than non-frontline workers and non-medical HCWs (41, 53, 55).

### Other Related Conditions (Distress, Psychological Trauma, and Burnout)

The studies in this review also reported additional outcomes such as distress, psychological trauma, and workplace burnout. High levels of workplace exhaustion and fatigue were prevalent (38, 42). Younger HCWs reported higher prevalence of symptoms of psychological trauma and distress compared to older HCWs (44, 48). Females, more than males, reported psychological trauma, burnout due to stressful work conditions, and distress (22, 29, 40, 59, 64) though one study reported greater psychological trauma symptoms among male HCWs (48). These sex differences also existed among physicians, with female physicians reporting higher burnout than their male counterparts (22, 43). Differences by HCW role were reported with nurses and healthcare assistants reporting greater distress than those in physician roles (44, 48). Nurses, trainees, and non-physicians reported also higher stress (20, 27, 59, 64). Among physician roles, resident physicians experienced greater burnout than attending physicians and physicians in branches of general surgery, gynecology, and orthopedics also reported higher burnout (22, 23). In terms of work duration, permanent workers reported greater burnout and fatigue compared to temporary workers (43).

## Discussion

Our findings highlight the high prevalence frequency of anxiety, depressive symptoms, insomnia, burnout, and distress that HCWs have experienced during the COVID-19 pandemic and are consistent with existing reviews (12, 65–70). Reviews published on this topic either cover shorter timeframes compared to this current review or report on studies only from China. Meta-analytic approaches used by some reviews may be subject to important methodological limitations. For instance, an earlier meta-analysis by Pappa et al. synthesized results from 13 studies to provide prevalence estimates of depression, anxiety, and insomnia (12). However, the paper also highlights limited data availability and important methodological sources of variation that contribute to between-study heterogeneity. Similar concerns around the lack of validated mental health outcome measures and appropriate comparisons to pre-outbreak time periods are also cited by Thombs et al. (71).

Our current review includes 51 studies with substantial variation in methodology and results reported which precluded the use of a meta-analytic approach and made a narrative review of results most suitable for this work. These studies employed a variety of instruments and used different cutoff thresholds to assess severity. Notably, the magnitude and severity of reported mental health outcomes may vary based on the validity and sensitivity of the measurement tools. The selection criteria of HCW samples also varied between studies, and HCWs' degree of exposure to patients with COVID-19 care also differed. It is likely that the degree of proximity to COVID-19 treatment may determine the extent to which HCWs experience grief, fear, anxiety, or concerns about their personal safety.

Some limitations are important to note. First, our search was conducted from December 2019 to June 2020 which covers ~7 months of the COVID-19 pandemic. Given the rapidly evolving literature in this area at present, it is possible that new studies may have been indexed later that we were unable to include in this review. Second, the timeline for this rapid review allowed for the search of only two electronic databases. Finally, there were very few studies from lower-middle income countries that could be used in our report indicating a need for more studies from these locations to improve the quality of available evidence.

Despite the methodological variation present in the studies reviewed, there are important themes that are predominant in the evidence thus far. Significant differential patterns by age, sex, and HCW role have emerged. Younger, female, non-physician HCWS, and frontline workers appear to be more vulnerable to poor mental health as seen in several studies reported above. Workplace burnout is also highly prevalent among these subgroups and is important to study further as it co-occurs with other mental health conditions such as depression (72). These differences based on demographics and work role are also observed in earlier reviews (12). It is important to note that the majority of non-physician HCWs are typically female, and it will be useful to disentangle the effects of sex and HCW role in future work. In our review, only 9 studies reported inclusion of frontline workers in their samples and 3 studies sampled non-physicians exclusively. Therefore, gaining a more comprehensive understanding of mental health in these subgroups will require additional studies. Nonetheless, such differences as seen in our review and elsewhere are likely to have varying consequences for HCWs in the larger context of workplace equity during major global health crises.

Support of HCW mental health in LMICs must be tailored from the perspective of these differences that could be potential harbingers of emerging disparities. These could likely place specific HCW subgroups (female HCWs, younger HCWs, non-physician workers) at a disproportionately higher risk for developing mental disorders. LMICs are known to experience challenges in investment in healthcare resources including inadequate personnel. Adaptations in healthcare delivery models such as repurposing of health facilities, task shifting without competency based trainings and supportive supervision, and redeployment from different departments to accommodate high patient volumes will inevitably place a greater burden on already vulnerable HCW subgroups. In addition to the burden of these stressors, HCWs are also likely to face economic changes such as exacerbated poverty and inequality that are projected to take place in LMICs due to the pandemic (73). Therefore, these have important implications for future research and intervention development. International institutes have developed guidelines to address mental health burden of HCWs. The United Nations and WHO published guidelines and recommendations on mitigating the mental health impact of COVID-19 on HCWs (74, 75). The National Academy of Medicine has also published strategies to support mental health of HCWs and clinicians during COVID-19 (76). In the context of the evidence presented above and by these major organizations, some general guidelines and recommendations are provided below in Tables 2A, B. These are aimed at improving both the generation of high quality evidence through research as well as intervention strategies to implement in practice for the support of HCWs' mental health in LMICs.

**Table 2A:** Mental health of HCWS during COVID-19: recommendations for research.

**Recommendations for Research on COVID related consequences on HCWs and mitigation strategies**
Better delineation of study samples–which worker roles are included or not and how those may impact study findings
Assess any reporting bias that may be due to data collection methods (e.g., online vs. in-person data collection)
Use of culturally informed screening instruments to assess mental health conditions
Addressing stigma-reduction on an urgent basis, intersectional nature of stigma, public health measures to educate populations about distress associated with increased stigma
Prospective assessment of samples to determine changes in duration and severity of symptoms
Continued examination of socioeconomic factors, including gender, race, and ethnic identity, that may affect mental health of HCWs
Continued examination of workplace factors that may influence the mental health of HCWs
Evaluation of intervention efficacy using appropriate measures based in evaluation science principles
Evaluation of policies and human resource management plans enacted to support HCWs and workplace mental health policy development and evaluation in LMICs and enhanced training, research and support to those working in humanitarian context

**Table 2B:** Mental health of HCWs during COVID-19: recommendations for policy and practice.

**Recommendations for policy and practice**
Addressing emerging disparities by worker role, age, sex, socioeconomic status, race, and ethnic identity; Policies focused on equity and more egalitarian workplace culture
Developing strategies to deal with protracted stress, grief, bereavement and using team science principles to improve organizational climate and leadership skills in managers and senior staff; routine assessment of stress, burn out and other mental health indicators and provision of timely, holistic support
Decision support system to help HCW address mental health and well-being of diverse at-risk populations and those with multimorbidity; inclusion of self-care modules to attend to their own stress and strains; especially enhanced support for the well-being and work performance of HCWs in humanitarian settings and those working directly with COVID-19 response and with key populations
Human rights-based and social justice services and training of HCWs who are embedded within ongoing emergency response
Development of brief, low intensity interventions addressing resilience, emergency preparedness and improved mental health outcomes, looking at short and long term mental health outcomes
Social and family needs of HCWs to be addressed at health systems level

## Conclusion

HCWs experience considerable adverse mental health outcomes in the context of COVID-19. Findings summarized in this review can inform approaches to monitoring and data collection for mental health outcomes among HCWs in LMICs. These findings can aid the tailoring of interventions and support strategies implemented at the institutional and national levels to mitigate and manage mental health conditions among HCWs in LMICs with a specific focus on vulnerable HCW subgroups.

## Author Contributions

MM, MR, and MK searched and screened studies. MM and MK extracted data from selected studies. MM wrote first draft of manuscript. All authors reviewed and edited manuscript.

## Conflict of Interest

The authors declare that the research was conducted in the absence of any commercial or financial relationships that could be construed as a potential conflict of interest.
